# Probing the *C*_3_ symmetry of gramicidin S

**DOI:** 10.1039/d6cb00134c

**Published:** 2026-06-19

**Authors:** Alex Hoose, Javier Garcia-Ruiz, Ciara C. M. Lally, Camilla Dondi, Maxim G. Ryadnov

**Affiliations:** a National Physical Laboratory Teddington Middlesex TW11 0LW UK max.ryadnov@npl.co.uk

## Abstract

Gramicidin S continues to inspire antibiotic designs. It exhibits a conserved two-fold (*C*_2_) symmetry postulated to underpin its biological activity. Here we probe the *C*_3_ symmetry of gramicidin S and its impact on the folding and antibacterial properties of this antibiotic.

## Abstract 2

Gramicidin S exhibiting *C*_3_ symmetry is reported.

## Graphical abstract

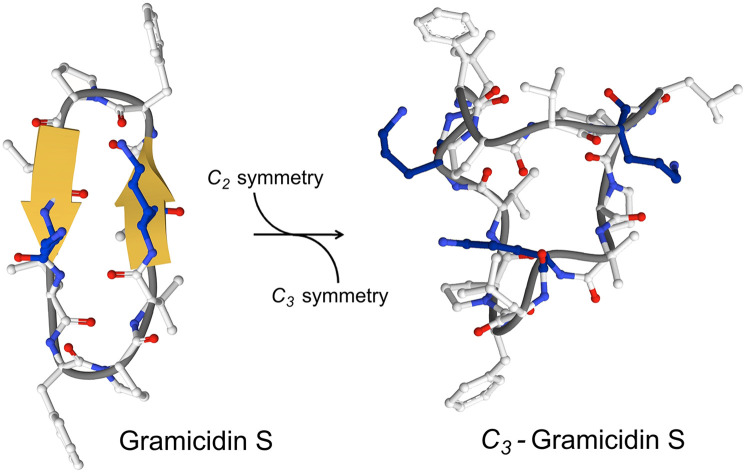

Gramicidin Soviet (GS) is a cyclopeptide antibiotic isolated from *Bacillus brevis*.^1^ GS disrupts microbial membranes by adsorption, *i.e.*, effectively but non-specifically,^2^ demonstrating the competitive exclusion principle applied to microbiota at the molecular level.^3–5^ Like other niche antibiotics GS is secreted by its producer host to outcompete microorganisms in the same environmental niche. This rationale is analogous to that of bacteriocins, which also disrupt membranes, but tend to target closely related bacteria.^6,7^ By contrast, GS does not appear to discriminate between Gram-positive and Gram-negative bacteria as well as fungi and red blood cells.^8^ This lack of selectivity is consistent with its role as a niche antibiotic rather than a drug candidate. However, due to its efficacy and speed of action, ability to kill dormant and resistant bacteria and inhibit biofilms, GS is being used as a molecular scaffold in search of new antibiotics for clinic.^9^

Most efforts to date have focused on improving the therapeutic index of the peptide balancing between its prominent membranolytic activity and poor selectivity. Approaches include sequence editing and sequence inversions, incorporation of non-proteinogenic amino acids and ring tuning in terms of size, rigidity and stereochemistry.^10–18^ Arguably, the most promising solutions involve the disruption of amphipathicity by single cationic residues introduced into the hydrophobic face of the peptide resulting in non-haemolytic versions of GS.^19^ This strategy also proves versatile for designing non-haemolytic antimicrobial peptides, in particular those of comparable sizes with GS.^20^ Similarly effective appear site-specific modifications with α,β-dehydroalanines,^21^ which are common in antibiotics such as nisin,^22^ and may provide an added value of stabilising GS against enzymatic degradation.

In one way or another, attempts to improve GS as a clinical antibiotic concern with its structural optimisations. The hallmark of GS is that it exhibits a conserved two-fold (*C*_2_) symmetry at both primary and secondary structure levels, which predefines its biological properties. The peptide comprises two copies of a pentapeptide Pro-Val-Orn-Leu-d-Phe which are linked into a continuous backbone. The head-to-tail cyclisation of the backbone gives rise to a symmetrical β-sheet of two antiparallel β-strands spaced by two type-II β-turns. This arrangement allows for the formation of a rigid, conserved decapeptide unit, stabilised by four intramolecular hydrogen bonds with hydrophobic and polar side chains placed on the opposite faces of the plane of the ring thereby forming an amphipathic structure (Fig. 1).^23–25^

**Fig. 1:**
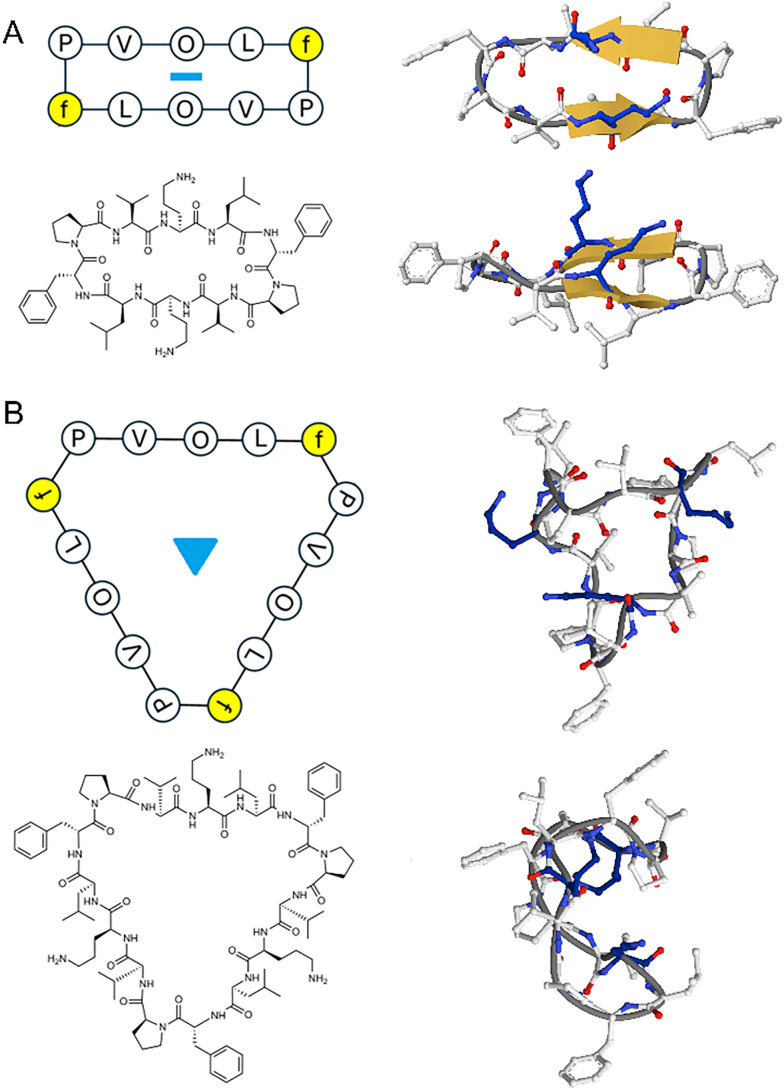
Peptide structure and design. Sequences shown as topology schematics (upper left), chemical structures (lower left) and minimised energy models (right) for (A) GS with a two-fold (*C*_2_) symmetry denoted by a blue rectangle and (B) 3GS with a three-fold (*C*_3_) symmetry denoted by a blue triangle. Yellow and white circles in topology schematics denote d and l amino acids, respectively. Computational models (right), face (upper) and side (lower) views, are energy minimised and structurally rendered (PDB 8RC7).^23–25^ The model highlight symmetry and amphipathicity in 3D, ornithine side chains (dark blue), nitrogen (light blue) and oxygen (red) of peptide bonds and β-strands (yellow arrows). The ribbon representations are superimposed on the stick presentations.

Existing evidence provides important insights into the folding traits of the antibiotic in reconstituted membranes.^26–28^ However, the structural optimisations of GS performed so far overlook symmetry considerations in the design of GS analogues. This is surprising given that symmetry is key for activity. Furthermore, rings of different sizes, without considering symmetry implications, were attempted by others to reveal that GS of 6 or 14 residues retain β-sheet conformations, whereas rings of 8, 12 and 16 residues give predominantly random coils.^17^ Although these designs may be viewed as having a *C*_2_-symmetry, none of them retains the original feature of the pentapeptide as a repeat unit, whilst all retain the ability to form intramolecular hydrogen bonds. Herein we probe the next, higher level of symmetry in GS to explore its impact on folding and antibacterial activity.

Our design rationale accepts the pentapeptide repeat unit as a symmetry integer in GS, with each unit separating two β-turns. Based on this rationale, a GS exhibiting a *C*_3_ symmetry combines three pentapeptide units intermitted with three β-turns into a triangular cyclopeptide, termed 3GS (Fig. 1). To probe the rationale, both GS versions were chemically synthesised in open and cyclised forms using conventional 9-fluorenyl-methoxycarbonyl (Fmoc) solid phase synthesis protocols (Fig. 1 and Fig. S1, S2 and Table S1). The open forms or linear sequences, GSO and 3GSO, were assembled on a 2-chlorotrytil resin which permits the cleavage of the assembled sequences with protecting groups kept intact and terminal amino and carboxy groups free. The backbone, head-to-tail cyclisation was then performed using an infinite volume strategy to give rise to the cyclised forms (Fig. S3). As expected, GS was found to exhibit appreciable antibacterial activities. Minimal inhibitory concentrations (MICs) for the peptide measured microdilution assays and automated analysis protocols^29^ were in the micromolar ranges typical of membrane active peptides (Table 1). GS is said to be preferentially active against Gram-positive bacteria, with varied activities against Gram-negative species. This tendency is comparable to that of bacteriocins such as epidermicin NI01 from *S. epidermidis*.^30^ MICs for both GS and NI01 were higher against Gram-negative bacteria (Table 1). Intriguingly, both GS and NI01 proved to be active against *A. baumanii* – a Gram-negative bacterium marked by WHO as a critical priority pathogen due to its high rate of resistance development.^31^ The results are consistent with the susceptibility of *A. baumanii* to membrane-active peptides. Polymyxin B – a membrane-active peptide used in clinic against Gram-negative bacteria – was found to be most effective against this and other Gram-negative bacteria but, as expected, ineffective against Gram-positive bacteria – the trend it shared with a *de novo* triskelion antimicrobial peptide (C_3+_) designed as a major component of antimicrobial capsids.^32^

**Table 1:** Minimal inhibitory concentrations of peptides used in the study

Bacteria^a^	Peptide (µM)						
	GS	3GS	GSO	3GSO	NI01^b^	PB^b^	C_3+_
*S. aureus* 29213	5	25	>50	>50	1.5	>50	>50
*E. coli* 25922	12.5	50	>50	25	>50	<1	1.5
*P. aeruginosa* 27853	25	>50	>50	>50	>50	1	3
*A. baumannii* 19606	3	3	>50	12.5	12.5	<1	nd

a Gram-negative bacteria are highlighted in grey.

b NI01 – epidermicin NI01, PB – polymyxin B; C_3+_ – *de novo* AMP.

Biological activities of GS and epidermicin result from their ability to lyse cell membranes. However, both these agents are folded in solution, which is in marked contrast to antimicrobial peptides (AMPs) that fold in response to membrane binding.^32–34^ Circular dichroism (CD) spectroscopy is a straightforward probe to monitor this property of AMPs in reconstituted membranes.^35–37^ Although GS has a distinctive CD spectrum, changes in peptide folding upon binding to membranes are more nuanced than appreciable.^26,28,37,38^ Indeed, differences in CD spectra obtained for the peptide in solution and in the presence of zwitterionic and anionic unilamellar vesicles used as reconstituted model membranes, were subtle (Fig. 2A).

**Fig. 2:**
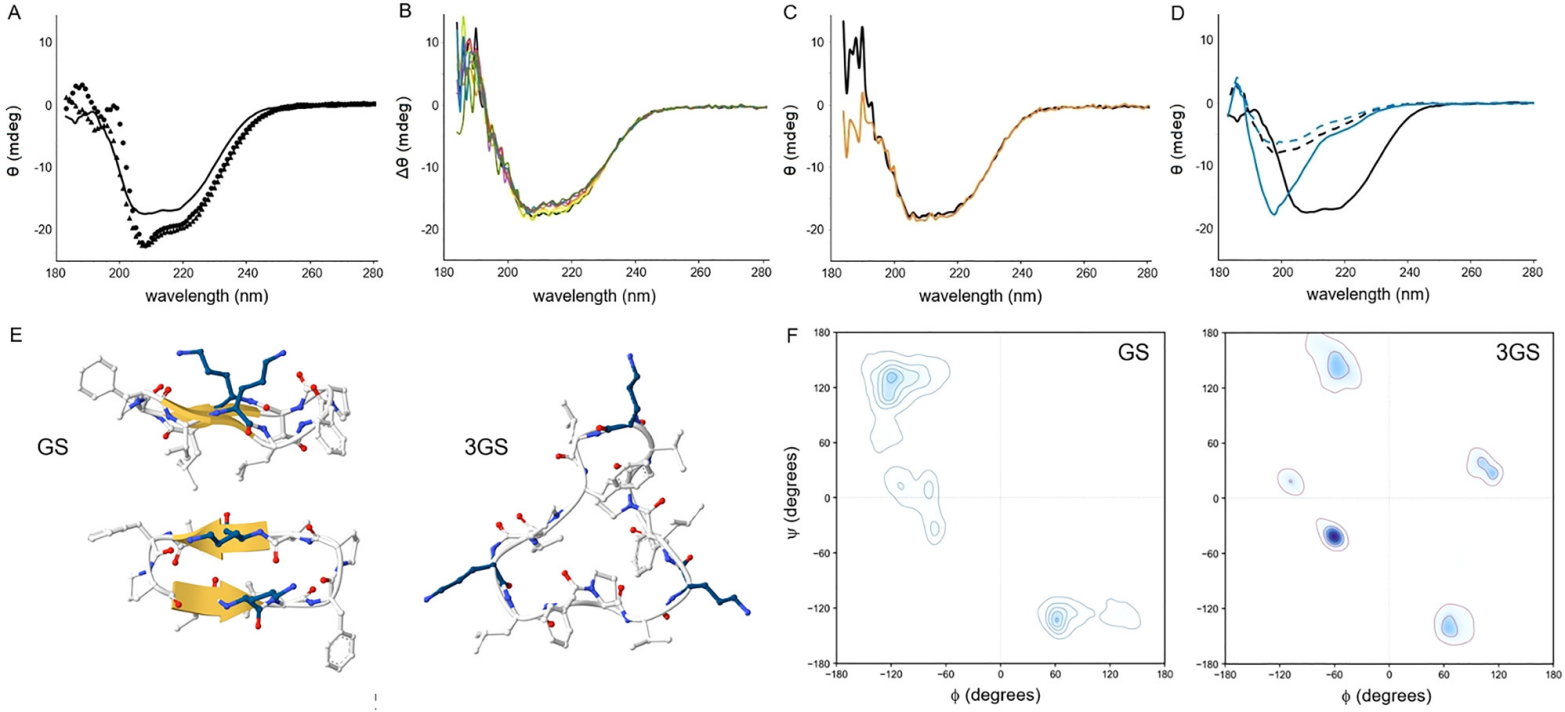
Peptide folding. (A) CD spectra for GS without (black line) and with membranes (POPC, black triangles; and POPC/POPG, black circles). (B) CD spectra for GS recorded every 10 °C (20–90 °C). (C) CD spectra for GS before (black) and after (orange) thermal denaturation. (D) CD spectra for GS (black), 3GS (blue), GSO (dashed black), 3GSO (dashed blue). Key: 50 µM peptide in 10 mM MOPS, pH 7.4. (E) 200 ns snapshots of atomistic MD simulations for GS (left, side and top views) and 3GS (right). Key: blue highlights ornithine side chains, red and light blue denote oxygen and nitrogen atoms, yellow denotes β-strands. (F) Ramachandran plots for GS (left) and 3GS (right).

The spectra obtained in solution contained characteristic features of GS, with a double minimum at around 206 and 219 nm, indicating electronic transitions π → π* and n → π*, respectively. Alongside with a less defined maximum at ∼190–195 nm, also corresponding to π → π* transitions, these signals suggest a helical structure. However, a typical α-helix would contain 208 and 222 nm minima, whereas a typical β-sheet signal has only a single minimum at 215 nm.

Thus, the peaks recorded for GS were blue shifted towards lower wavelengths, when compared to helical signals, which can be attributed to conformational constraints set by cyclisation. No changes were observed in the spectral features when GS was subjected to thermal denaturation (Fig. 2B). The fold did not undergo conformational transitions up to 90 °C. There is an indication of a decreasing signal-to-noise ratio in the thermal denaturation spectra, which can be due to differential absorption flattening because of the tendency of GS molecules to associate or clump together.^28^

However, CD spectra recorded before and after melts, followed by cooling, were identical (Fig. 2C). These results show that GS adopts an optimally stable conformation in solution. By contrast, in membranes the minima shifted to 208 nm and a relatively amplitude at around 225 nm (n → π*). Interestingly, both these changes reflect the conformational behaviour of type II β-turns in GS. The negative band at 225 nm is characteristic of type II β-turns, whilst the range of 200–210 nm is more reflective of changes in β-turns.^37^

Furthermore, upon binding π → π* transitions become more intense, which was found in both membrane types with a stronger negative intensity at 208 nm and an emerging maximum at 200 nm, indicative with a positive Cotton effect. Typical of β-turns, being more pronounced in the presence of membranes these signals suggest that β-turns mediate the binding of GS to membranes. Indicative of a mixture of β-sheet and β-turn conformers, the shifts appeared as subtle conformational adjustments rather than apparent changes, which is analogous to bacteriocins, which like GS are folded in solution.^7^ Although the exact reason for helical features observed in the CD spectra of GS has yet to be unambiguously determined, such a phenomenon can relate to the ability of GS to form helical double-stranded, twisted β-sheet channels which may offer a mechanistic insight into the biological activity of GS.^39,40^ An additional insight comes from the differences found in the minima ratios ([*θ*]_219_/[*θ*]_206_*versus* [*θ*]_222_/[*θ*]_208_) that tended to decrease for GS upon membrane binding from ∼0.95 to 0.8. Such decreases, which are common for GS and its analogues in different membrane systems,^16,37,39^ suggest that in membranes GS is likely to exist as an isolated molecule rather than form an oligomer. Unlike α-helices, whose backbones favour intermolecular interactions readily forming membrane permeabilising assemblies,^41,42^ β-type folds on membranes may be restricted to intramolecular interactions promoting membrane adsorption instead.^37^

With no apparent differences seen between the CD spectra of GS in both membrane types (Fig. 2A), this restriction agrees with that the biological activity of GS is predetermined by its *C*_2_ symmetry. The symmetry sets up intramolecular hydrogen bonding which appears to maintain in aqueous and lipid environments alike. Modifications in amphipathicity and β-turns lead to variations in folding but do not appear to impact on the symmetry, which is preserved in a rather substantial repertoire of GS analogues to date. Many of these have comparable biological profiles, but none offers a clinically superior candidate, suggesting that the restriction of the hydrogen bonding translates to that of biological activity. In other words, if the symmetry is kept, which ensures biological activity, structural modifications fall short of notably improving the antibiotic performance of GS. In this light, 3GS comprises all the structural attributes of GS, except *C*_2_ symmetry. The *C*_3_ symmetry retains the pentapeptide units separated by β-turns, amphipathicity, a net positive charge and the cyclopeptide backbone, but is expected to disrupt the intramolecular hydrogen bonding characteristic of GS. Thus, 3GS provides an ultimate test for the rationale of GS as a *C*_2_ symmetry antibiotic *versus* a cyclopeptide exhibiting the structural characteristics of antimicrobial peptides.

CD spectra for 3GS were indicative of type-II β-turns with a characteristic minimum at ∼195 nm and a maximum at ∼185 nm. The spectra contained elements of random coil conformations but lacked features that could be assigned to β-sheets or α-helices (Fig. 2D). In accord with this, β-turns in 3GS appeared to be more prevalent in membranes, with CD spectra exhibiting a dominant minimum at 225 nm. Like GS, the band indicates that the turns become stabilised in the membrane environments.^37^ Unlike GS, stronger binding of 3GS could be ascertained to the anionic membranes. As expected for cationic, amphipathic peptides which do not adopt a stable fold in solution, this was further supported by comparable CD spectra for GSO and 3GSO (Fig. S4A–C). Thermal denaturation experiments revealed that the conformation was unstable, with appreciable flattening of the minimum at ∼195 nm at elevated temperatures. Upon cooling, the conformation failed to recover indicating an irreversible conformational transition (Fig. S4D and E). The post-melt spectral signature of 3GS was comparable to that of both open forms, 3GSO and GSO, which were nearly identical (Fig. 2D and Fig. S4E). These results indicate that the pentapeptide units did not fold into a stable secondary structure in 3GS and the open forms, whereas the hydrogen bond formation was constrained to preserve β-turns.

Molecular dynamics (MD) simulations concurred with the experimental results in that GS maintained its structure with no appreciable changes observed over 200 ns (Fig. 2E and Fig. S5 and Video S1). The simulations also revealed apparent rotational flexibility for tyrosine side chains, which impacted on the conformational stability of 3GS, but not on that of GS (Videos S1 and S2). In both peptides, β-turns remained fixed, suggesting that the introduction of an additional pentapeptide unit in *C*_3_ disrupts the hydrogen bonding supporting β-sheet formation in GS, consistent with the loss of a stable structure upon transition from *C*_2_ to *C*_3_ symmetry (Fig. 2E and Fig. S5 and Videos S1, S2). Reflecting these differences, Ramachandran plots were found to reveal antiparallel β-sheets and type II’ β-turns for GS, whereas type II and type II’ β-turns without β-sheets were evident for 3GS (Fig. 2F).^43^

As a likely consequence, 3GS had no appreciable activity against the bacteria used, except for *A. baumanii*, for which MICs for 3GS and GS were same and within one microdilution of the MIC for polymyxin B. Since 3GS is a larger molecule than GS, the increase in size may compensate for the loss of the structure responsible for the activity. However, MICs for 3GSO were three times higher for *A. baumanii*, whereas GSO showed no activity against any bacterium used (Table 1). The findings indicate that the lack of biological activity of the open forms directly correlates with that of their folding propensities. The findings also suggest that 3GS can selectively target bacteria susceptible to membrane-active antibiotics, and that *C*_3_ symmetry, as opposed to the mere extension of GS by one pentapeptide unit, is essential for this. In this regard, MIC variations between GS and 3GS against Gram-negative *E. coli* and *P. aeruginosa* were most apparent. These may not necessarily indicate underlying differences in the mode of action but rather reflect impact of experimental conditions on killing kinetics. MICs derive from optical density measurements taken overnight for the bulk culture, which makes them subject to inoculum effects, with no considerations for changes at the cellular level, which renders a comparative assessment incomplete.^44–46^

Therefore, we sought additional evidence from single-cell experiments within the first hour of antibacterial treatment. As gauged by live-cell imaging, both bacteria were comparably resistant against GS and 3GS within the first hour of incubation. This is despite that both peptides were used at the same concentration (20 µM), which for GS was a dilution higher or comparable with the MICs, and a dilution lower for 3GS (Fig. 3 and Fig. S6 and S7). By contrast, activity trends for NI01, which was found to be inactive, and C_3+_*de novo* peptide, which was found to be most active, against these Gram-negative bacteria, were consistent with the corresponding MICs, except the atypical activity of NI01 against *P. aeruginosa*. At peptide concentrations well beyond MIC values (150 µM), killing rates by NI01, GS and 3GS were comparable, but with up to 40% of cells remaining viable. The observed effects directly relate to the mode of action of membrane-active peptides, which are most effective against bacterial cells within their first doubling time (20–30 min). Longer experimental incubations are subject to inoculum effects and peptide depletion due to irreversible complexation with phospholipids.^44–48^ A complementary scenario, particularly relevant to *P. aeruginosa*, concerns with the formation of cell-deficient forms such as spheroplasts, which are mediated by membrane-active antibiotics.^46,49^

**Fig. 3:**
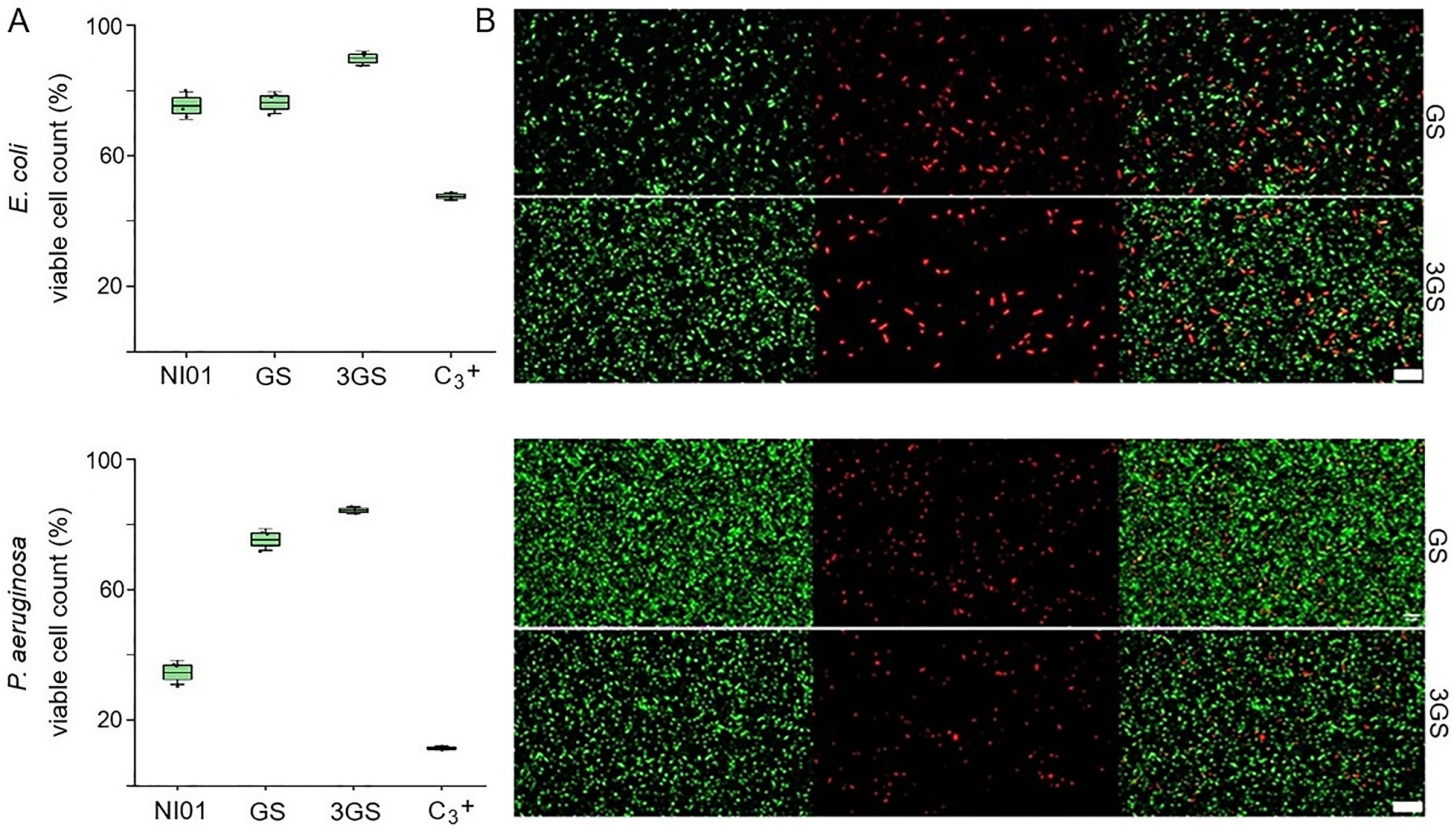
Bacteria cell viability. (A) total viable counts of bacterial cells grown over 60 min with antibacterial agents – epidermicin NI01, GS, 3GS and C_3+_*de novo* AMP. Boxplots: solid horizontal lines denote mean and whiskers denote standard deviation. The data is overlaid as dots and presented as the percentage of the total counts of viable bacteria in the total number of cells with standard deviations for three independent biological replicates (*n*  =  3). Total number of untreated cells is taken as 100%. The total number of cells treated with 70% (v/v) aqueous ethanol is taken as 0%. (B) Fluorescence micrographs of bacterial cells treated with GS and 3GS and stained with Syto9 (green) and propidium iodide (PI, red), with images highlighting viable cells (left), dead cells (middle), and viable and dead cells merged (right). Scale bars are 20 µm.

Collectively, the results prompt several conclusions. First, the transition from the *C*_2_- to *C*_3_-symmetry carries the cost of losing the rigid, amphipathic β-sheet structure naturally optimised for the broad-spectrum antibiotic activity of GS. The introduction of an additional pentapeptide unit into GS drives the other two pentapeptide units apart thereby disrupting the hydrogen bonding underpinning β-sheet formation. This contrasts with GS analogues comprising 15 residues whose pentapeptide units are merely extended to hepta- and octa-peptide units, which allows them to retain the original *C*_2_ symmetry and antibacterial activity.^50^ Second, and consequently, the biological activity of GS becomes impaired, rather than lost, and presents an opportunity for the tuneable targeting of bacteria susceptible to membrane-active antibiotics. Of relevance this can be to applications where strong, broad-spectrum antibiotics are not desirable, or the eradication of a particular bacterium or strain in an environmental niche hosting other bacteria is preferred. In this regard, 3GS may hold promise for the design of narrow-spectrum antibacterial agents. Third, the backbone cyclisation to install the *C*_3_-symmetry in GS remains necessary to elicit antibacterial activity. Forth, the impaired antibacterial activity for 3GS, especially against Gram-positive bacteria, may result from it being a larger molecule without a defined structure in solution which may hinder its passing through peptidoglycan, lipoteichoic acid and lipopolysaccharide layers that tend to entrap larger molecules.^32,51^ Fifth, implicitly, this study supports the notion of GS adopting higher order helical structures, which bears relevance to supramolecular antimicrobial designs such as self-assembling β-hairpin and β-helical motifs.^52,53^

Although this study did not aim to solve the exact mechanism of action for GS, our findings may have mechanistic implications for membrane disruption likely caused by the peptide in line with multi-modal mechanisms observed for membrane-disrupting peptides as well as with the earlier evidence of antimicrobial β-structures assembling into nanoscale pores.^7,20,54–57^ Surprisingly, despite the popularity of GS in antibiotic research, there is only circumstantial evidence as to the ability of the peptide to form pores in membranes.^10,58–63^ In this light, follow-up mechanistic studies in reconstituted and live membranes for GS and its analogues can prove crucial for a qualitatively more efficient rationale in the design of GS-inspired antibiotics, and as a quantitative exemplar in the current efforts to trace protein sequence to activity.^64^

## Author contributions

Alex Hoose: conceptualization, methodology, investigation, data analysis, writing – original draft, review and editing. Javier Garcia-Ruiz: methodology, investigation, data analysis, writing – review and editing. Ciara C. M. Lally: methodology, investigation, data analysis, writing – review and editing. Camilla Dondi: methodology, investigation, data analysis, writing – review and editing. Maxim G. Ryadnov: conceptualisation, supervision, methodology, data analysis, project administration, funding acquisition, writing – original draft, review and editing.

## Conflicts of interest

There are no conflicts to declare.

## Supplementary Material

CB-007-D6CB00134C-s001

CB-007-D6CB00134C-s002

CB-007-D6CB00134C-s003

## Data Availability

The data supporting this article has been included as part of the supplementary information (SI). Supplementary information: Table S1, Fig. S1–S7, Videos S1 and S2, experimental methods for peptide synthesis, biophysics, MD simulations and biological assays. See DOI: https://doi.org/10.1039/d6cb00134c.
