# Supplementary material for: Probing the C3 symmetry of gramicidin S
Source: RSC Chem Biol. 2026 Jun 19. Online ahead of print. doi: 10.1039/d6cb00134c (PMC13307312; doi:10.1039/d6cb00134c)
Supplement: CB-OLF-D6CB00134C-s001 [file CB-OLF-D6CB00134C-s001.pdf]

Supplementary Information (SI) for RSC Chemical Biology.
This journal is © The Royal Society of Chemistry 2026

Supporting Information

Probing the C_3 symmetry of Gramicidin S

Alex Hoose,[a] Javier Garcia-Ruiz,[a] Ciara C. M. Lally,[a] Camilla Dondi[a] and Maxim G. Ryadnov[a,*]

[a]National Physical Laboratory, Teddington, Middlesex, TW11 0LW, United Kingdom

Videos, Table and Figures

Video S1 – 200 ns atomistic simulation for GS

Video S2 – 200 ns atomistic simulation for 3GS

Table S1. Characteristics of the peptides used in the study.

name	peptide	sequence	topology	secondary structure	m/z	
					calc	found
GS	Gramicidin S	cyclo(VOLfPVOLfP)	cyclopeptide	β-sheet and β-turn	1141.71	1141.71
GSO	GS open form	VOLfPVOLfP	linear	β-turn	1159.72	1159.72
3GS	C_3 GS analogue	cyclo(VOLfPVOLfPVOLfP)	cyclopeptide	β-turn	1712.06	1712.06
3GSO	3GS open form	VOLfPVOLfPVOLfP	linear	β-turn	1730.07	1730.07

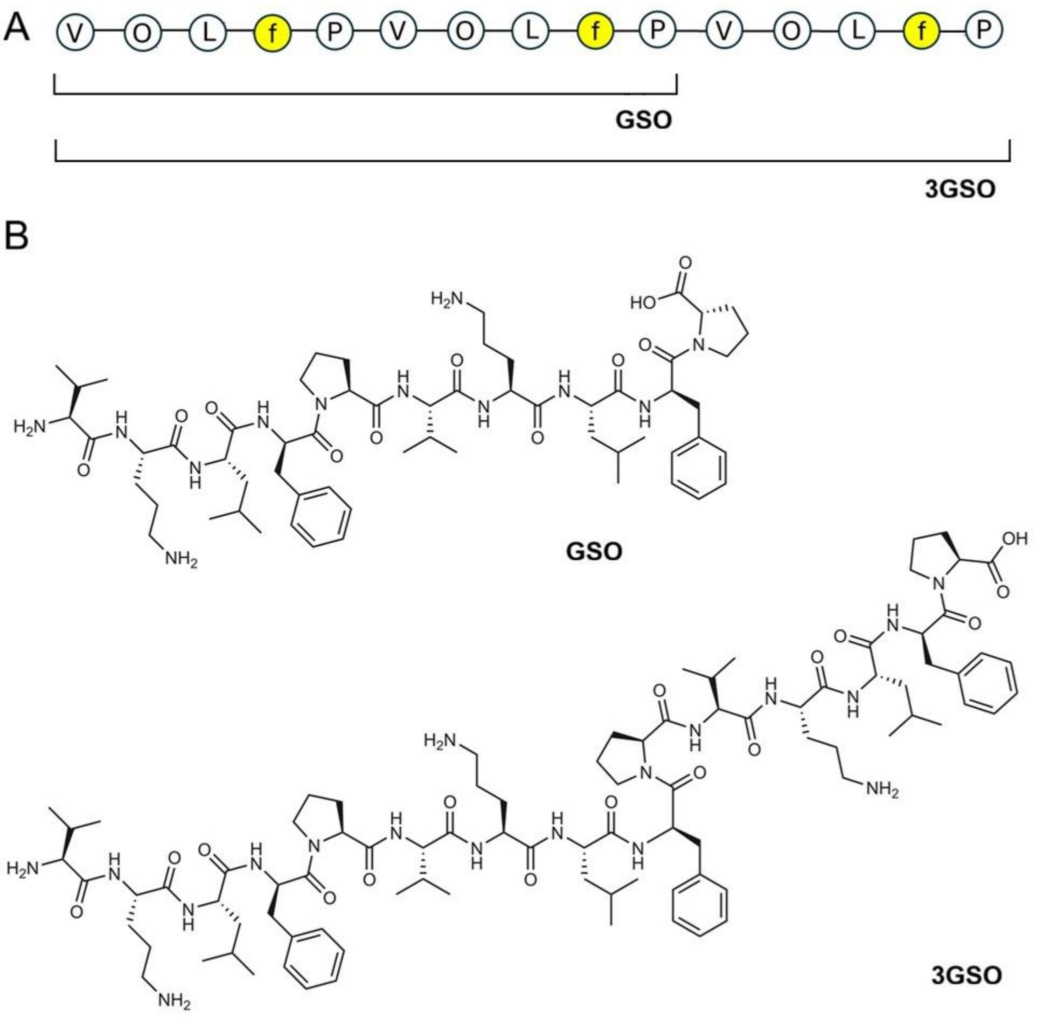

Figure S1. GS open forms (linear sequence) shown as topology schematics (A) and chemical structures (B). Yellow and white circles denote D and L amino acids, respectively.

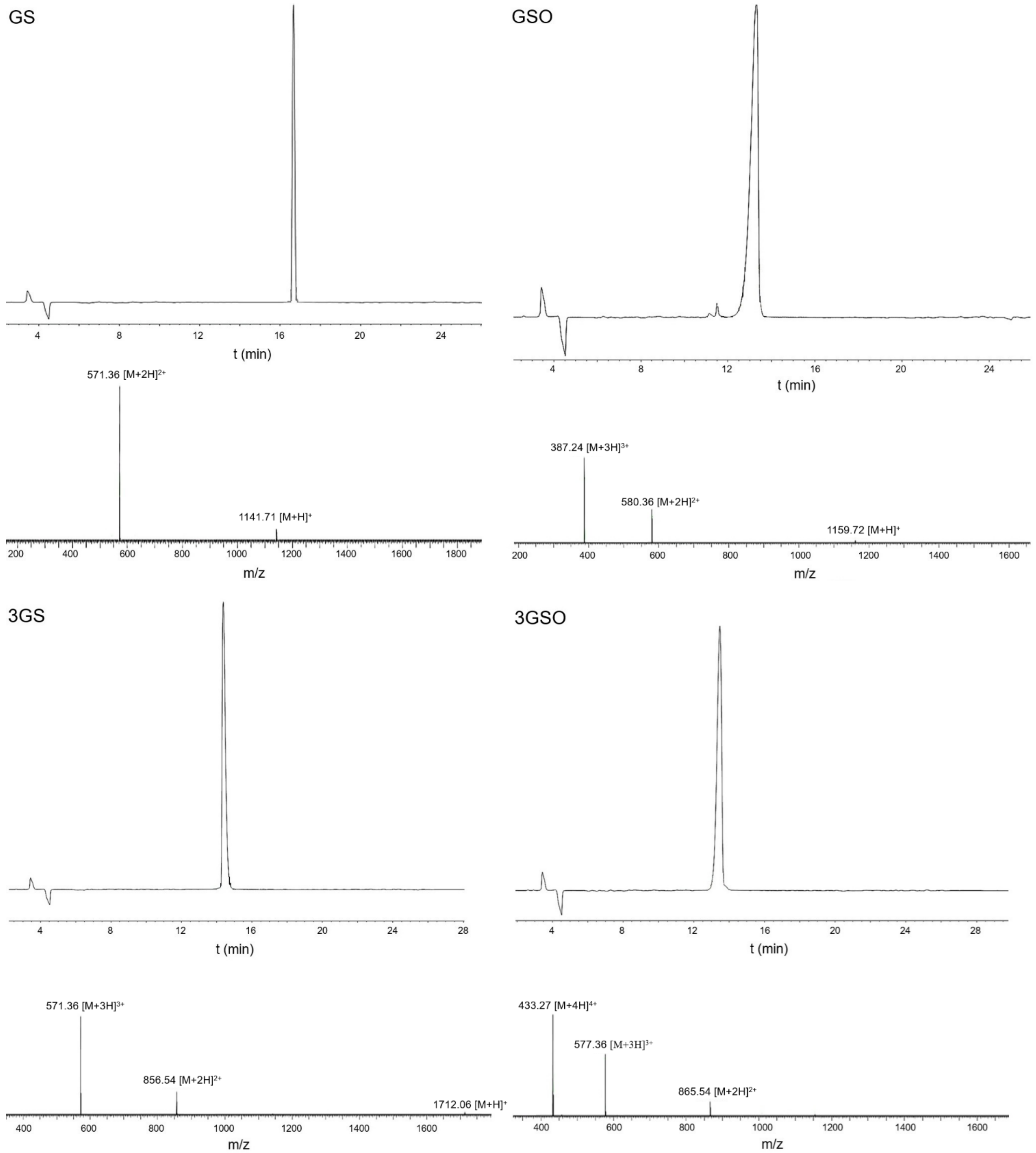

Figure S2. Analytical RP-HPLC profiles (upper) and LC-MS spectra (lower) for the peptides used in the study.

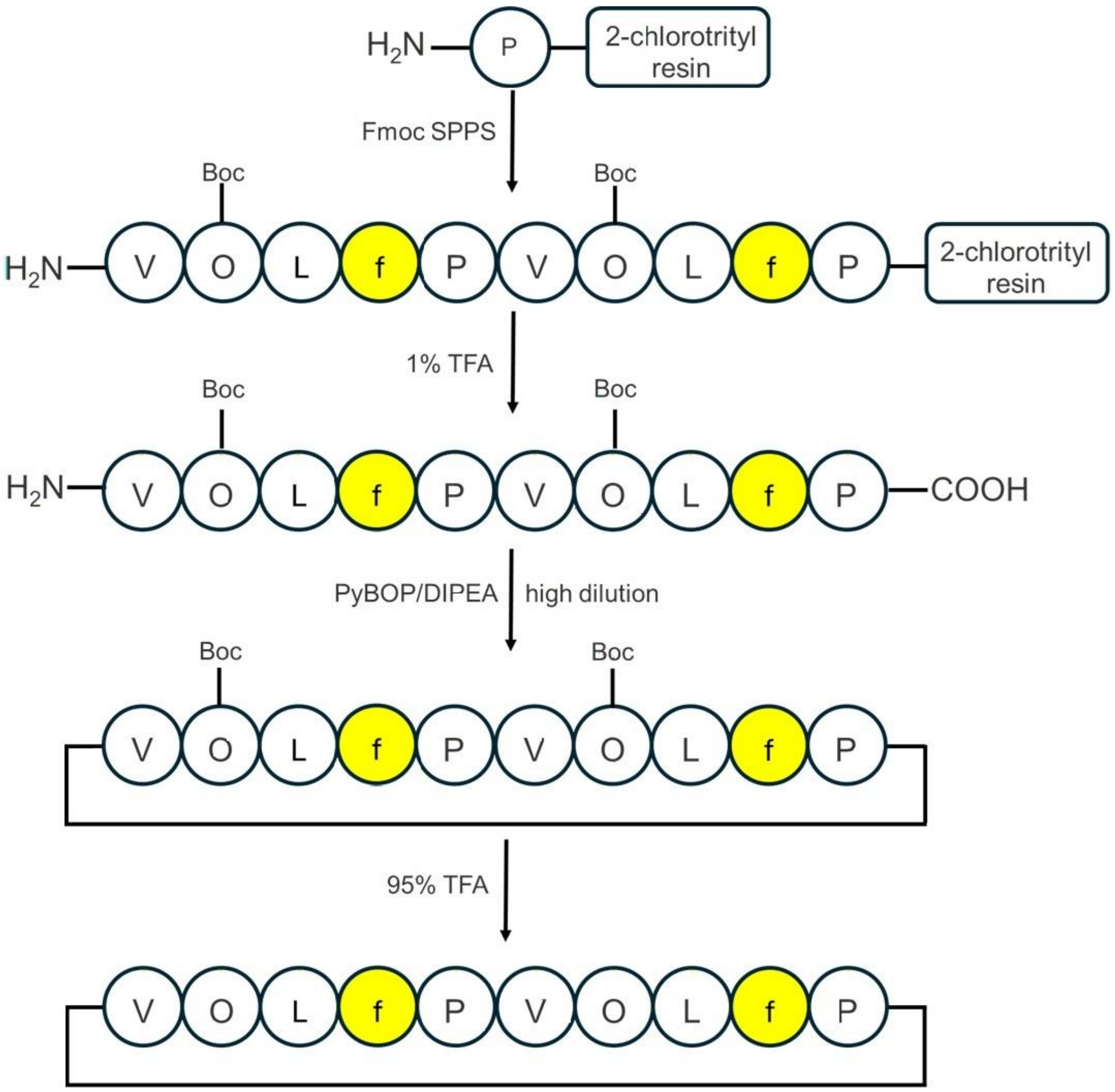

Figure S3. Schematic representation of the synthetic route used to synthesise GS. The same strategy was used for 3GS. Yellow and white circles denote D and L amino acids, respectively.

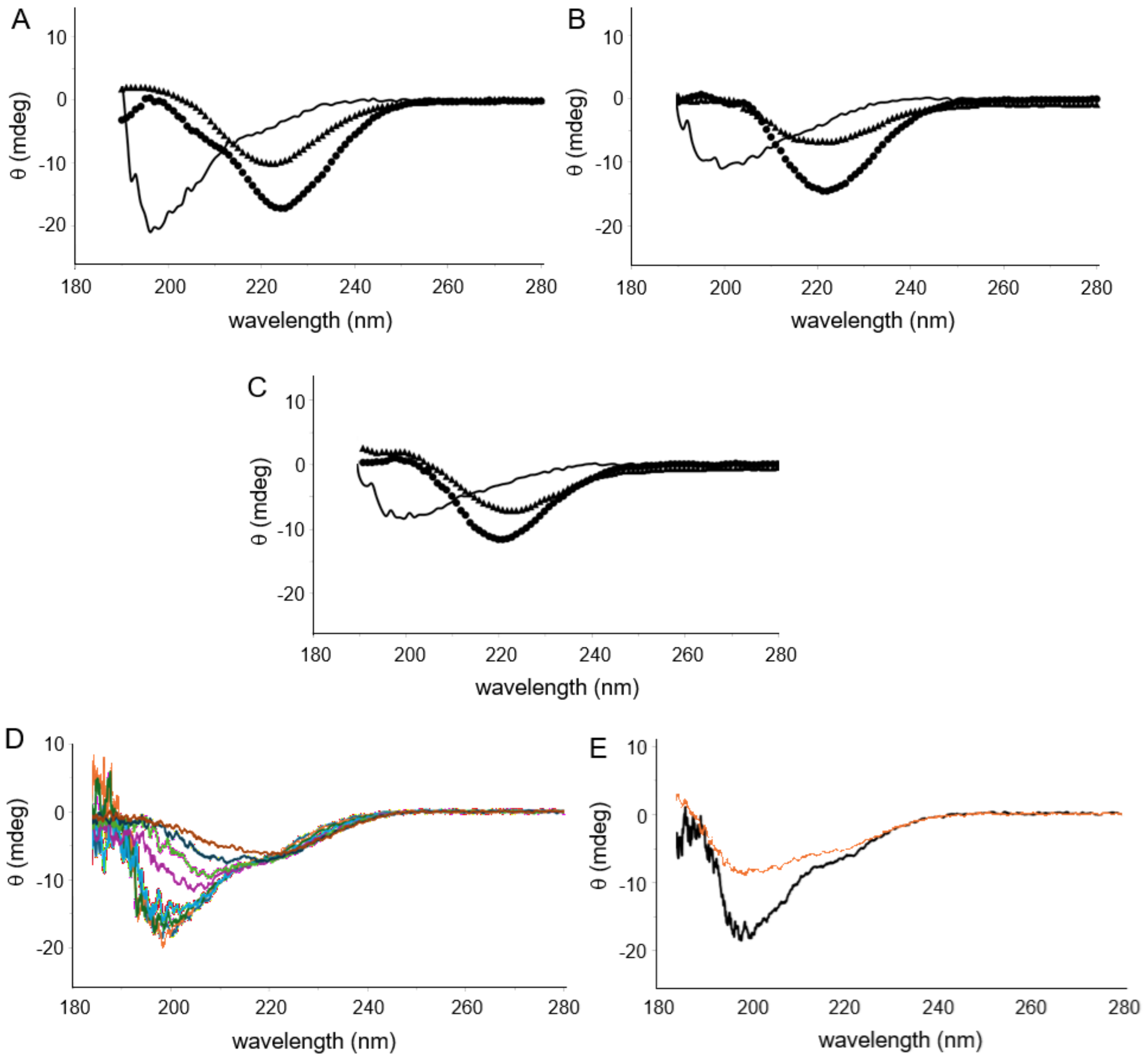

Figure S4. Peptide folding. CD spectra for 3GS (**A**), GSO (**B**), 3GSO (**C**) without (black line) and with membranes (POPC, black triangles; and POPC/POPG, black circles). (**D**) CD spectra recorded at for 3GS as a function of temperature (20-90°C) recorded every 10°C. (**E**) CD spectra for 3GS before (black line) and after (orange line) thermal denaturation. Key: 50 µM peptide in 10 mM MOPS, pH 7.4.

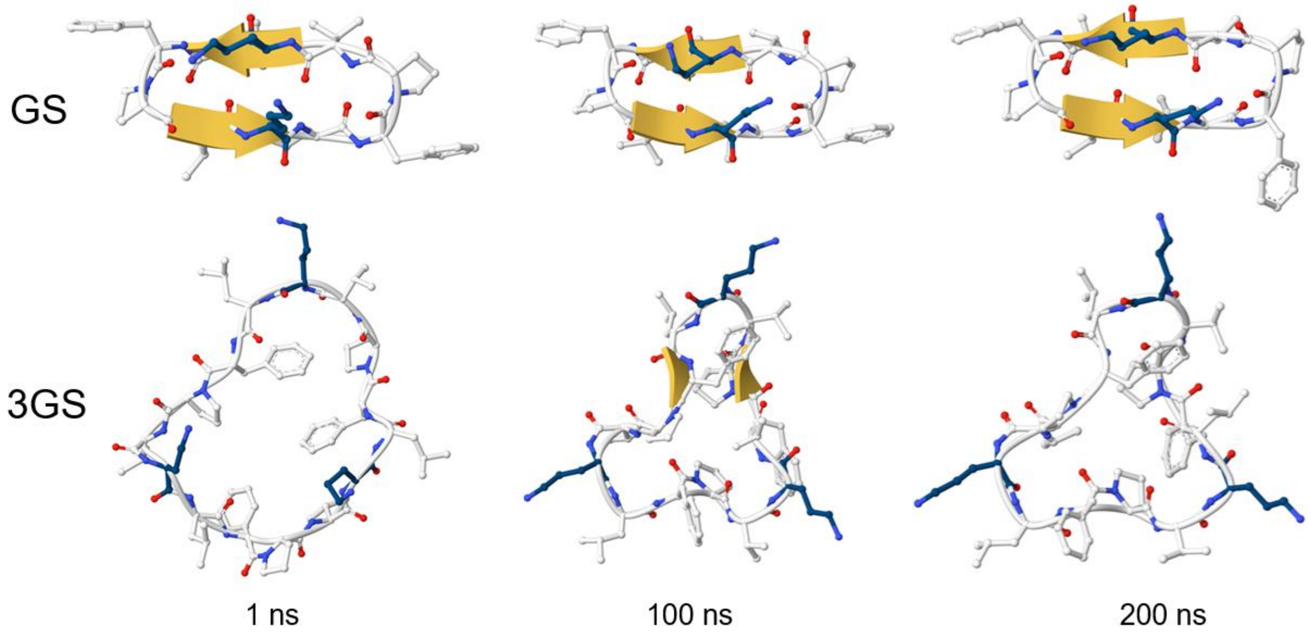

Figure S5. Snapshots of atomistic MD simulations (Videos S1 and S2) for GS and 3GS: after initial equilibrium phase (1 ns), half through the simulation (100 ns) and the resulting simulation (200 ns). Key: blue highlights ornithine, red and light blue denote oxygen and nitrogen, yellow denotes β-strands.

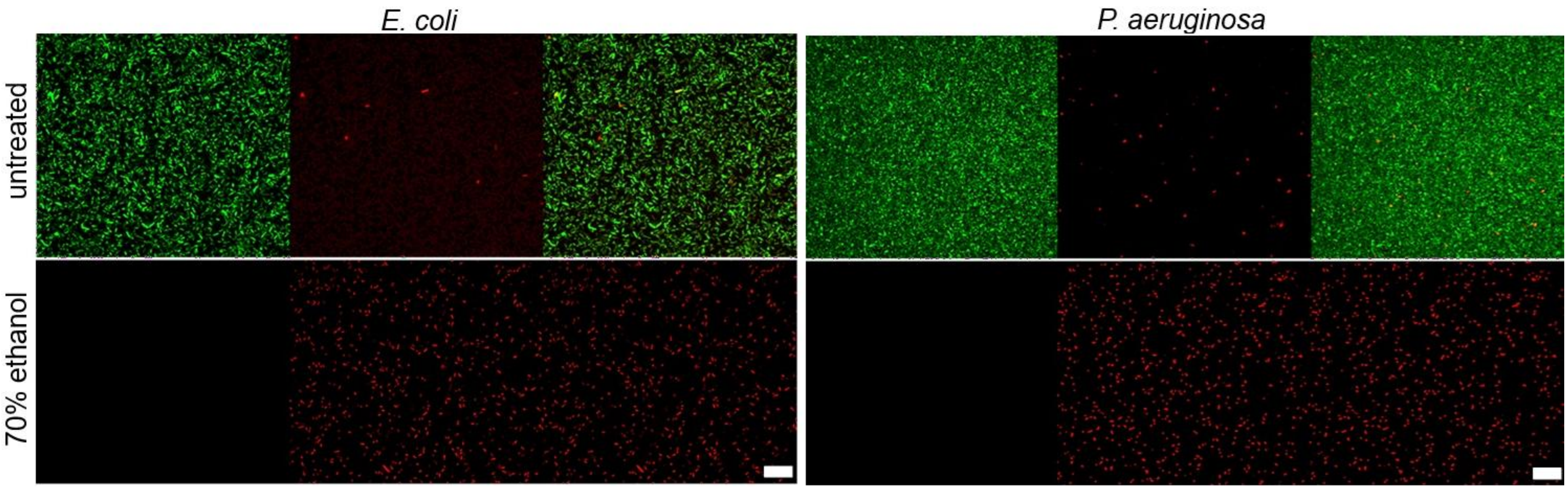

Figure S6. Bacterial cell viability. Fluorescence micrographs of background controls - 100% viability (untreated cells) and 0% viability (cells treated with 70% aq. Ethanol). Cells were stained with Syto9 (green) and propidium iodide (PI, red), with images highlighting viable cells (left), dead cells (middle), and viable and dead cells merged (right). Scale bars are 20 μm.

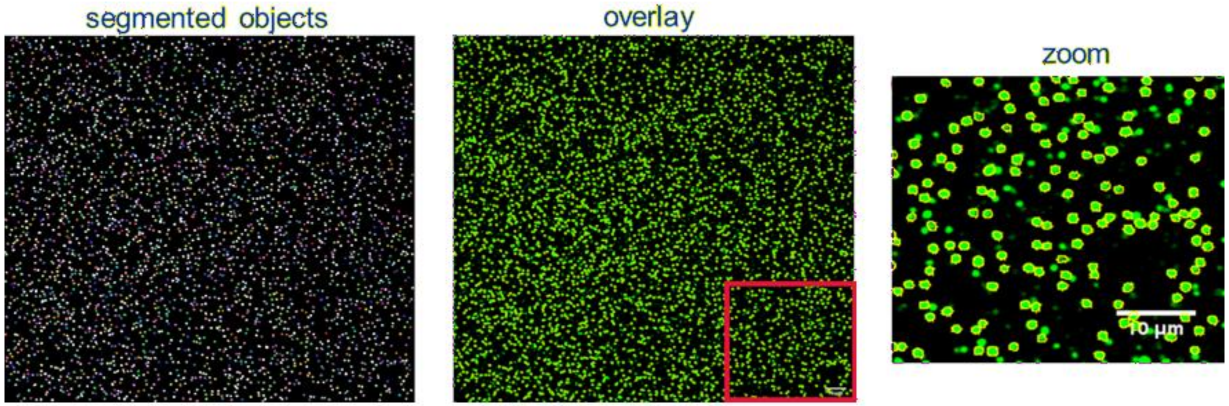

Figure S7. Bacterial cell viability. An exemplar for image segmentation of fluorescence micrographs by StarDist. The plugin creates a mask of segmented objects for both live and dead channels separately, counts are averaged over three images acquired. Scale bar is 10 μm.

Experimental

Materials and reagents. Fmoc α-amino protected amino acids, H-Pro-2-Cl-Trt Resin, benzotriazol-1-yloxytripyrrolidinophosphonium hexafluorophosphate (PyBOP), *N,N*-diisopropylethylamine (DIPEA), pyridine and 3-morpholinopropane-1-sulfonic acid (MOPS) were purchased from Sigma Aldrich. Trifuoroacetic acid (TFA), Triisopropylsilane (TIS), *N,N*-Diisopropylcarbodiimide (DIC) and Oxyma Pure were from Fluorochem. Piperidine, formic acid and HPLC grade TFA were from Alfa Aesar. *N,N*-dimethylformamide (DMF), diethyl ether, acetonitrile, methanol and dichloromethane (DCM) were from Rathburn. 1-palmitoyl-2-oleoyl-glycero-3-phosphocholine (POPC) and 1-palmitoyl-2-oleoyl-sn-glycero-3-phospho-(1'-rac-glycerol) (POPG) were from Avanti Polar Lipids (Alabaster, USA). All bacteria were from ATCC. Polymixin B was purchased from Fisher Bioreagent. Mueller Hinton media broth was from Oxoid, and LIVE/DEAD^{TM} BacLight^{TM} bacteria viability kit was from ThermoFisher.

Peptide synthesis. A Liberty Blue microwave peptide synthesizer (CEM) was used to assemble peptide sequences. NI01 and C_3^+ triskelion were made as described elsewhere.[7, 32] GS and GS-derived peptides were assembled on a H-Pro-2-chlorotrityl resin following conventional Fmoc/^{t}Bu solid-phase synthesis protocols. The resin (loading = 0.27 mmol g^{-1}, m = 0.37 g, n = 0.1 mmol) was swollen in DMF (1 x 15 mL x 30 min) prior to synthesis. DIC/OxymaPure were used as coupling reagents, with double coupling at 25 °C (2 x 25 min) performed throughout the synthesis. Amino acids were used at a stoichiometric ratio of 5 to the amino component for each coupling, with coupling reagents used at 10 (DIC) and 5 (Oxyma Pure) ratios. The completed sequences for GS and 3GS were cleaved from the resin using 1 % TFA / DCM (10 x 10 ml x 2 min), with sidechain protecting groups kept intact. Each cleavage aliquot was immediately neutralized with 10 % pyridine / MeOH (10 x 2 ml), and the resulting aliquots were combined with resin washings performed on the resin with DCM (3 x 30 ml x 2 min), MeOH (3 x 30 ml x 2 min), DCM (2 x 30 ml x 2 min), MeOH (3 x 30 ml x 2 min). The combined mixtures were subjected to evaporation under reduced pressure, and the resulting solution (< 2 ml) was precipitated (1 x 10 ml) and washed with ice cold water (3 x 10 ml), prior to desiccation for 16 h. The protected sequences (1 eq.) were dissolved in DMF under high dilution conditions (c = 0.5 mM); prior to the addition of PyBOP (2 eq.), DIPEA (5 eq.) and Oxyma (2 eq.). The cyclization was carried out stirring at 25 °C for 16 h, followed by DMF evaporation at 40 °C. The cyclized peptides were then precipitated by Et_2O (40 ml), spun down (3700 rpm, 100 mm radius rotor, 15 min), lyophilized and treated with a cleavage cocktail (94 % TFA, 3 % TIS, 3 % water, 5 mL) while agitating for 150 min. The mixture evaporated, and the peptide was precipitated (Et_2O, 40 mL) and spun down (3700 rpm,

100 mm radius rotor, 15 min). The obtained pellet was then washed (Et_2O, 2 x 40 mL), dissolved in acetonitrile (20 ml), diluted into water (30 ml) and lyophilized. GS0 and 3GSO were cleaved from resin via this approach.

The peptides were purified in a reversed phase high-pressure liquid chromatography (RP-HPLC) system (Waters Prep LC150) using C18 columns (30 mm x 250 mm, 130 Å, 5 µm) at a flow rate of 40 mL/min using a 20-60% buffer B gradient over 40 min (buffer A is 5% and buffer B is 95% aqueous CH_3CN, each containing 0.1% TFA) at 214 nm.

The fractions of the purified peptides were evaporated and analysed using RP-HPLC to confirm the purity of 95-99% and using high-resolution liquid chromatography mass spectrometry (LC-MS, Thermo Scientific Q-Exactive equipped with a HESI probe):

MS $[M+H]^+$: GS, *m/z* 1141.71 (calculated), 1141.71 (observed); GSO, *m/z* 1159.72 (calculated), 1159.72 (observed); 3GS, *m/z* 1712.06 (calculated), 1712.06 (observed).

MS $[M+2H]^{2+}$: GS, *m/z* 571.36 (calculated), 571.36 (observed); GSO, *m/z* 580.36 (calculated), 580.36 (observed); 3GS, *m/z* 856.54 (calculated), 856.54 (observed); 3GSO, *m/z* 865.54 (calculated), 865.54 (observed).

MS $[M+3H]^{3+}$: GSO, *m/z* 387.24 (calculated), 387.24 (observed); 3GS, *m/z* 571.36 (calculated), 571.36 (observed); 3GSO, *m/z* 577.36 (calculated), 577.36 (observed).

MS $[M+4H]^{4+}$: 3GSO, *m/z* 433.27 (calculated), 433.27 (observed).

Circular dichroism (CD) spectroscopy. CD spectra were recorded on a JASCO J-1500 spectropolarimeter fitted with a Peltier temperature controller. The measurements were taken in ellipticities in mdeg at stated concentrations in 10 mM MOPS (pH 7.4), and in POPC or POPC/POPG (3:1, molar ratio) in 10 mM MOPS at a lipid-peptide ratio of 100. The data was collected with a 0.1 nm step, 1 sec collection time per step, and is presented as the average of 4 scans. Thermal denaturation spectra were recorded at 208 nm, with 1°C intervals using 1 nm bandwidth, 180 s equilibration time for each spectrum and with 2 °C/min ramp rate, spectra recorded at every 10°C are presented in Figures 2 and S4.

Lipid vesicle preparation. Unilamellar vesicles were assembled from POPC, to mimic zwitterionic mammalian membranes, and from POPC mixed with POPG at 3:1 molar ratios, to emulate anionic bacterial membranes. The lipids were stored at -20°C as supplied and before use were equilibrated to room temperature. The lipids were weighted up and then dissolved in chloroform. The preparations (1 mL, 5 mg/mL) were vortexed for 20 sec, evaporated under a nitrogen flow for 60 min to form a dry thin film, which was hydrated in 10 mM MOPS buffer (pH 7.4), vortexed for 2 min and bath sonicated for 30 min at 0°C. The obtained suspension was extruded using a hand-held extruder (Avanti Polar

lipids) (polycarbonate filter, 0.05 μm) to give a clear solution of unilamellar vesicles adjusted to 1-2 mg/mL.

Photon correlation spectroscopy. Unilamellar vesicles were screened for homogeneity and size in quartz low volume cuvettes or folded capillary cells at 25°C using Zetasizer Ultra Red (Malvern Instruments Ltd, Worcestershire, UK). Hydrodynamic radii values were obtained through the fitting of autocorrelation data using Zetasizer Software (ZS Explorer), with a mean of two independent preparations with each measurement consisting of a minimum of 10 recordings.

Minimum inhibitory concentration (MIC) measurements. MIC values were obtained using broth microdilutions on *E. coli ATCC 25922, P. aeruginosa ATCC 27853*, *S. aureus ATCC 29213* and *A. baumanii ATCC 19606* following the guidelines of the Clinical and Laboratory Standards Institute, BS EN ISO 20776-1:2020 and the established protocols of analysis.[26] Briefly, 100 μL of (0.5–1) × 10^6 CFU per mL of each bacterium in Mueller Hinton media broth was incubated in 96-well microtiter plates with 100 μL of serial two-fold dilutions of peptides (from 100 to 0 μM) at 37 °C on a 3D orbital shaker. The absorbance was measured at 600 nm using a SpectraMax i3x Multi-Mode microplate reader (Molecular Devices). MICs were defined as the lowest peptide concentration that inhibited visible bacterial growth after 24 h at 37 °C. All measurements were done in triplicates, and results are summarized in Table 1.

Bacterial growth inhibition. Bacteria were treated for 60 min with peptide at the stated concentrations (10 mM MOPS, pH 7.4) at room temperature. Bacteria treated with 70% (v/v) aq. ethanol for 60 mins provided positive controls (0% viability). Untreated cells (cells treated with the buffer only) provided negative controls (100%). Bacteria were stained using the LIVE/DEAD™ BacLight™ according to manufacturer's protocols before placing in Ibidi chambers (Thistle Scientific, UK) for imaging.

Confocal fluorescence microscopy. Bacteria were imaged using a confocal laser scanning microscope (Stellaris 5, Leica) using a 63×/1.4 NA objective with 504/523 nm and 549/615 nm for Syto9 and propidium iodide, respectively. Images (3D stacks) were processed using ImageJ software and bacteria cells were counted for live (green) and dead (red) using a deep-learning-based method for segmentation (StarDist, Fiji plugin).

Chemical structures and computational models. All chemical structures were produced using ChemDraw Professional (version 18.2.1.50) and presented as produced without rearrangements of bonds or stereochemistry. The computational models are based on PDB entry 8RC7,[23] were energy minimised (Avogadro, ver. 1.2.0)[24] and structurally rendered (Mol*).[25] For 3GS, the structure was

drawn in ChemDraw and exported as a SMILES string for energy minimisation and structural rendering.

Molecular dynamics simulations. Initial configurations for peptides were prepared using the CHARMM-GUI web-based graphical interface (http://www.charmm-gui.org)[S1,S2], a CHARMM-modified TIP3P water model[S3] was used to solvate peptide into a cubic box. To avoid periodic boundary artifacts a minimum distance of 10 Å was maintained between peptide atoms and box borders. Potassium (K^+) and chloride (Cl^-) ions were used to neutralize the net charge of each simulation box and establish a physiological salt concentration of 0.15 M. All MD simulations were executed using GROMACS 2019.3,[S4] and the CHARMM36m forcefield (charmm36-feb2026_cgenff-5.0.ff)[S5] was used to describe peptides with parameters for non-standard amino acids including ornithine and D-phenylalanine. Particle-Mesh Ewald (PME)[S6] and the LINCS algorithm[S7] were employed to compute long-range electrostatics and constrain bonds involving hydrogen atoms to their correct lengths after an unconstrained update. The Verlet cut-off scheme was used for neighbour searching. The cut-offs for both Lennard-Jones and Coulomb potentials were set to 12 Å, with the Lennard-Jones potential smoothly transitioned to zero using a force-switch function between 10 and 12 Å. The potential energy in every simulated case was minimized using 5000 steps of the steepest-descent algorithm to ensure that a maximum force value (tolerance) is smaller than 1000 kJ mol^{-1} nm^{-1}. The position restraints were applied to heavy atoms of the peptide backbone (400 kJ mol^{-1} nm^{-2}) and sidechains (40 kJ mol^{-1} nm^{-2}). Following energy minimization, equilibration was performed in a two-stage protocol. First, an NVT equilibration phase was conducted using the Nose-Hoover thermostat (NHt)[S8, S9] over 125 ps at 303.15 K, the time integration step of 1 fs and the same positional restraints as the minimization step. Second, NPT equilibration was performed for 125 ps using the Parrinello-Rahman barostat (PRb)[S10] to couple the pressure to 1 bar isotropically (τ_p = 5.0 ps, compressibility = 4.5×10^{-5} bar^{-1}), at 303.15 K (NHt) with the time integration step of 1 fs. At this stage, dihedral restraints (force constant = 4.0 kJ mol^{-1} rad^{-2}) were applied alongside position restraints to preserve the initial peptide backbone geometry. PRb was used to control pressure during both the NPT equilibration and production phases. For the final production stage, unrestrained MD simulations were run over 200 ns in the NPT ensemble at 1 bar and 303.15 K with the time integration step of 2 fs (NHt, τ_t = 1.0 ps, and PRb, τ_p = 5.0 ps). Post-processing of the resulting trajectories was carried out (GROMACS) to remove water molecules and counterions. To eliminate sidechain-induced rotational and translational drift and ensure accurate structural comparisons, a least-squares fitting of backbone atoms was applied to the initial configuration. Visualizations were rendered using Mol* (https://molstar.org/),[S11] with structural analyses done using MDAnalysis (v2.10.0).[S12]

References

S1. Jo, S., Kim, T., Iyer, V. G., & Im, W. *J. Comput. Chem.*, 2008, **29**, 1859–1865.

S2. Lee, J., Cheng, X., Swails, J. M., Yeom, M. S., Eastman, P. K., Lemkul, J. A., Wei, S., Buckner, J., Jeong, J. C., Qi, Y., Jo, S., Pande, V. S., Case, D. A., Brooks, C. L., MacKerell, A. D., Klauda, J. B., & Im, W. *J. Chem. Theory Comput.* 2015, **12**, 405–413.

S3. Jorgensen, W. L., Chandrasekhar, J., Madura, J. D., Impey, R. W., & Klein, M. L. *J. Chem. Phys.* 1983, **79**, 926–935

S4. Abraham, M. J., Murtola, T., Schulz, R., Páll, S., Smith, J. C., Hess, B., & Lindahl, E. *SoftwareX*, 2015, **1-2**, 19–25.

S5. Huang, J., Rauscher, S., Nawrocki, G., Ran, T., Feig, M., de Groot, B. L., Grubmüller, H., & MacKerell, A. D. *Nat. Methods*, 2016, **14**, 71–73.

S6. Essmann, U., Perera, L., Berkowitz, M. L., Darden, T., Lee, H., & Pedersen, L. G. *J. Chem. Phys.* 1995, **103**, 8577–8593.

S7. Hess, B., Bekker, H., Berendsen, H. J. C., & Fraaije, J. G. E. M. *J. Comput. Chem.*, 1997, **18**, 1463–1472.

S8. Nosé, S. *Mol. Phys*. 1984, **52**, 255–268.

S9. Hoover, W. G. *Phys. Rev. A*, 1985, **31**, 1695–1697.

S10. Parrinello, M., & Rahman, A. *J. Appl. Phys.* 1981, **52**, 7182–7190.

S11. Sehnal, D., Bittrich, S., Deshpande, M., Svobodová, R., Berka, K., Bazgier, V., Velankar, S., Burley, S. K., Koča, J., & Rose, A. S. *Nucleic Acids Res.* 2021, **49**, W431–W437.

S12. Michaud-Agrawal, N., Denning, E. J., Woolf, T. B., & Beckstein, O. *J. Comput. Chem.*, 2011, **32**, 2319–2327.
